# Causal relationship between gut microbiota and Behçet’s disease: a Mendelian randomization study

**DOI:** 10.3389/fmicb.2024.1416614

**Published:** 2024-06-12

**Authors:** Yu-Nan Li, Tong Chen, Yang Xue, Jun-Ya Jia, Tie-Kun Yan, Peng-Cheng Xu

**Affiliations:** ^1^Department of Nephrology, Tianjin Medical University General Hospital, Tianjin, China; ^2^Department of Hematology, Tianjin Medical University General Hospital, Tianjin, China

**Keywords:** gut microbiome, Behçet’s disease, causality, Mendelian randomization, autoimmune disease (AD), genome-wide association study

## Abstract

**Background:**

While observational epidemiological studies have suggested an association between gut microbiota and Behçet’s disease (BD), the causal relationship between the two remains uncertain.

**Methods:**

Statistical data were obtained from gut microbiome Genome-Wide Association Studies (GWAS) published by the MiBioGen consortium, and genetic variation points were screened as instrumental variables (IV). Mendelian randomization (MR) study was performed using inverse variance weighted (IVW), weighted median, MR-Egger regression, simple mode, and weighted mode methods to evaluate the causal relationship between gut microbiota (18,340 individuals) and BD (317,252 individuals). IVW was the main method of analysis. The stability and reliability of the results were verified using the leave-one-out method, heterogeneity test, and horizontal genetic pleiotropy test. Finally, a reverse MR analysis was performed to explore reverse causality.

**Results:**

Inverse variance weighted (IVW) results showed that the genus Parasutterella (OR = 0.203, 95%CI 0.055–0.747, *p* = 0.016), Lachnospiraceae NC2004 group (OR = 0.101, 95%CI 0.015–0.666, *p* = 0.017), Turicibacter (OR = 0.043, 95%CI 0.007–0.273, *p* = 0.001), and Erysipelatoclostridium (OR = 0.194, 95%CI 0.040–0.926, *p* = 0.040) were protective factors against BD, while Intestinibacter (OR = 7.589, 95%CI 1.340–42.978, *p* = 0.022) might be a risk factor for BD.

**Conclusion:**

Our study revealed the causal relationship between gut microbiota and BD. The microbiota that related to BD may become new biomarkers; provide new potential indicators and targets for the prevention and treatment of BD.

## 1 Introduction

Behçet’s disease (BD) presents as a rare systemic vasculitis ailment typified by oral aphthous ulcers, genital ulcers, mucocutaneous lesions, and ocular involvement and it is one of the most common causes of blindness (Zeidan et al., 2016; Leccese and Alpsoy, 2019). BD is a great public health concern; however, the etiology of BD remains elusive, with immune system dysregulation believed to play a contributory role in its onset (Takeuchi et al., 2015).

Gut microbiota (GM) is a group of widely distributed, and diverse microbial communities intricately involved in human metabolism and immune regulation. Increasing evidence underscores the association between gut microbiota and the pathogenesis of numerous diseases, including metabolic disorders, autoimmune conditions, and malignancies (Maynard et al., 2012; Bishehsari et al., 2020; Fan and Pedersen, 2021; Rasouli-Saravani et al., 2023). Likewise, recent research has highlighted the connection between gut microbiota and BD. An imbalance in gut microbiota composition and function may be involved in the etiology and progression of BD (Consolandi et al., 2015; Shimizu et al., 2016a). However, the causal relationship between gut microbiota and BD remains incompletely understood, given the presence of reverse causality and confounding factors.

Mendelian randomization (MR) is a genetic method to determine the causal relationship between exposure and disease outcomes (Emdin et al., 2017). Given that alleles are randomly assigned and fixed before birth, they are relatively independent of the effects of environmental factors and are determined prior to the onset of disease, thus addressing the limitations of traditional observational studies that are affected by confounding factors and reverse causation (Smith and Ebrahim, 2003; Davies et al., 2018; Hartwig et al., 2018). In the present study, we employed MR to investigate the potential causal effects of gut microbiota on BD, aiming to offer a more robust foundation of evidence-based medical support for their relationship.

## 2 Materials and methods

### 2.1 Study design

**Figure 1:**
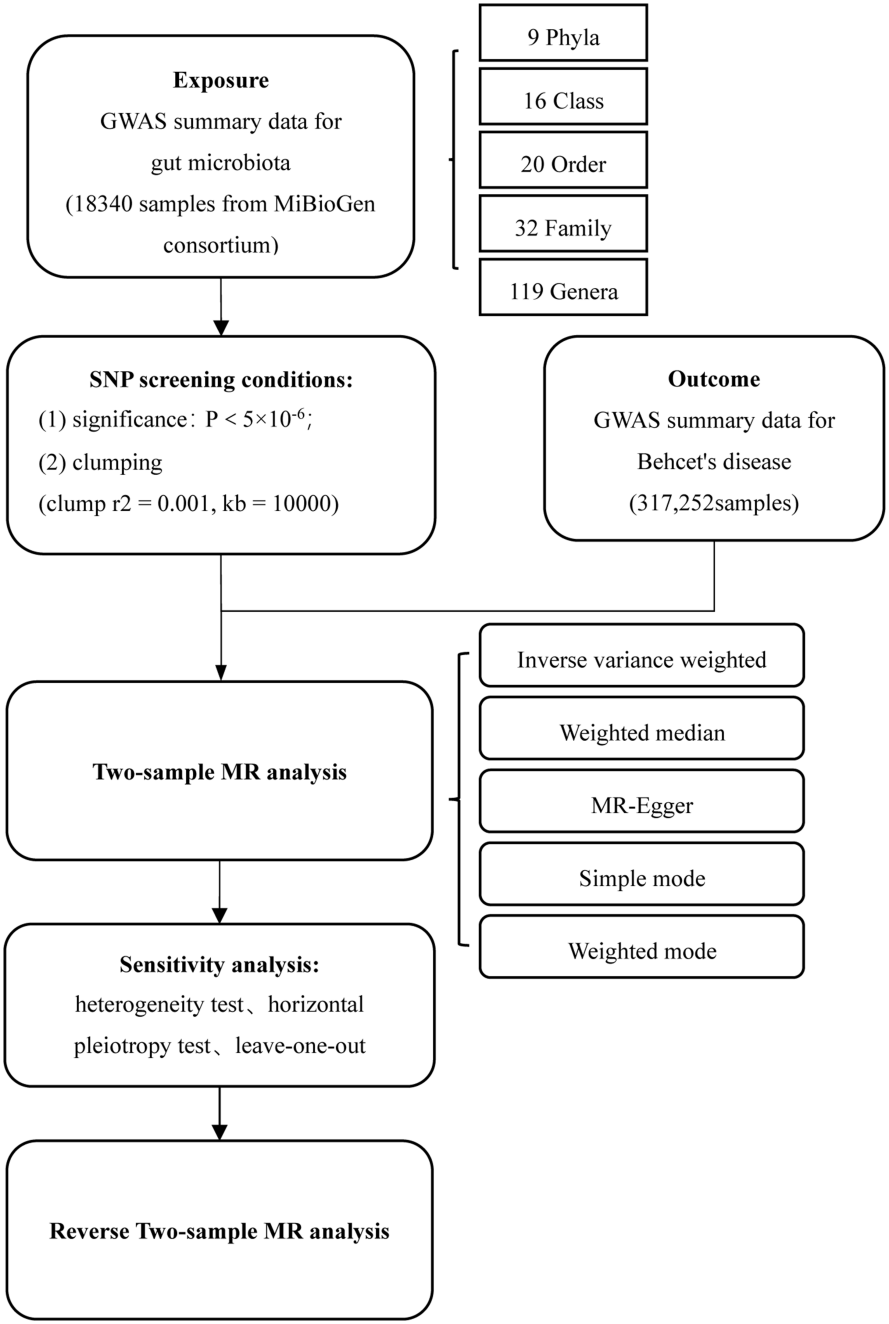
Flowchart of MR analysis. MR, Mendelian randomization; and SNP, Single nucleotide polymorphism.

Figure 1 depicts the flow chart detailing the MR analysis. In this study, single nucleotide polymorphisms (SNPs) significantly associated with exposure factors were employed as instrumental variables (IVs). The IVs satisfy three conditions: (1) the IVs should be significantly associated with gut microbiota; (2) the IVs should be independent of confounders; and (3) the IVs only affect the outcome events solely through exposure factors.

### 2.2 Data sources

The summary statistics of the human gut microbiome used in this study were obtained from the Genome-Wide Association Study (GWAS) meta-analysis conducted by the MiBioGen consortium (Kurilshikov et al., 2021), with a total sample size of 18,340 individuals from 24 cohorts, encompassing 5,747,754 SNPs. Genetic data for BD were obtained from GWAS study by Sakaue et al. (2021), containing 19,084,009 SNPs with a sample size of 317,252.

### 2.3 Identification of instrumental variables

Fifteen unspecified gut microbiota were excluded, resulting in 196 bacterial taxa included in the subsequent MR analysis, comprising 9 phyla, 16 classes, 20 orders, 32 families, and 119 genera.

Significant SNPs closely associated with exposure were selected from the GWAS pooled data, and SNP loci with *p* < 5 × 10^−6^ were chosen considering that there were few SNP loci with *p* < 5 × 10^−8^ in gut microbiota. To mitigate bias caused by linkage disequilibrium (LD) among SNPs and to ensure the independence of genetic variation, we employed *r*^2^ value of 0.001 and a window size of 10,000 kb. SNPs with strong linkage disequilibrium (*R*^2^ > 0.8) replaced those that did not existed in the GWAS data of exposure and outcome. The *F*-statistic (BETA2/SE2) was used to assess the strength of IVs, if the corresponding *F*-statistic >10, it was considered that there was no bias of instrumental variables. SNPS with *F* ≤ 10 were screened out and palindromic SNPs were excluded during the analysis.

### 2.4 Statistical analysis

In this study, we utilized the “Two Sample MR” package in R software (version 4.3.2) for conducting bidirectional two-sample MR analysis. The inverse variance weighted (IVW) was employed as the main MR method to explore the causal relationship between the gut microbiome and BD. Additionally, to enhance the reliability of our findings, we conducted supplementary analyses using MR-Egger, weighted median, simple mode, and weighted mode methods.

Sensitivity analysis includes heterogeneity test, horizontal pleiotropy test, and leave-one-out. In this study, we used Cochran’s *Q* test to gauge heterogeneity, where a *p* value <0.05 signified the existence of heterogeneity. When faced with heterogeneity, we referred to the random-effects IVW as the final MR result (Bowden et al., 2018). For the detection of horizontal pleiotropy in SNPs, we employed the MR Egger intercept test, considering *p* < 0.05 as indicative of possible horizontal pleiotropy (Bowden et al., 2015; Verbanck et al., 2018). Furthermore, we conducted the leave-one-out analysis to test the stability of results.

## 3 Results

### 3.1 Causal effects of gut microbiota on BD

After screening the GWAS data of gut microbiota, a total of 1,496 independent SNPs (*p* < 5 × 10^−6^) were identified, spanning five taxonomic levels: phylum, order, family, and genus. Ultimately, 35 SNPs were selected as instrumental variables, with the genus Parasutterella accounting for 11 SNPs, the genus Lachnospiraceae NC2004 group for three SNPs, the genus Turicibacter for six SNPs, the genus Erysipelatoclostridium for eight SNPs, and genus Intestinibacter for seven SNPs. Notably, all *F*-statistics for these SNPs exceeded 10.

**Figure 2:**
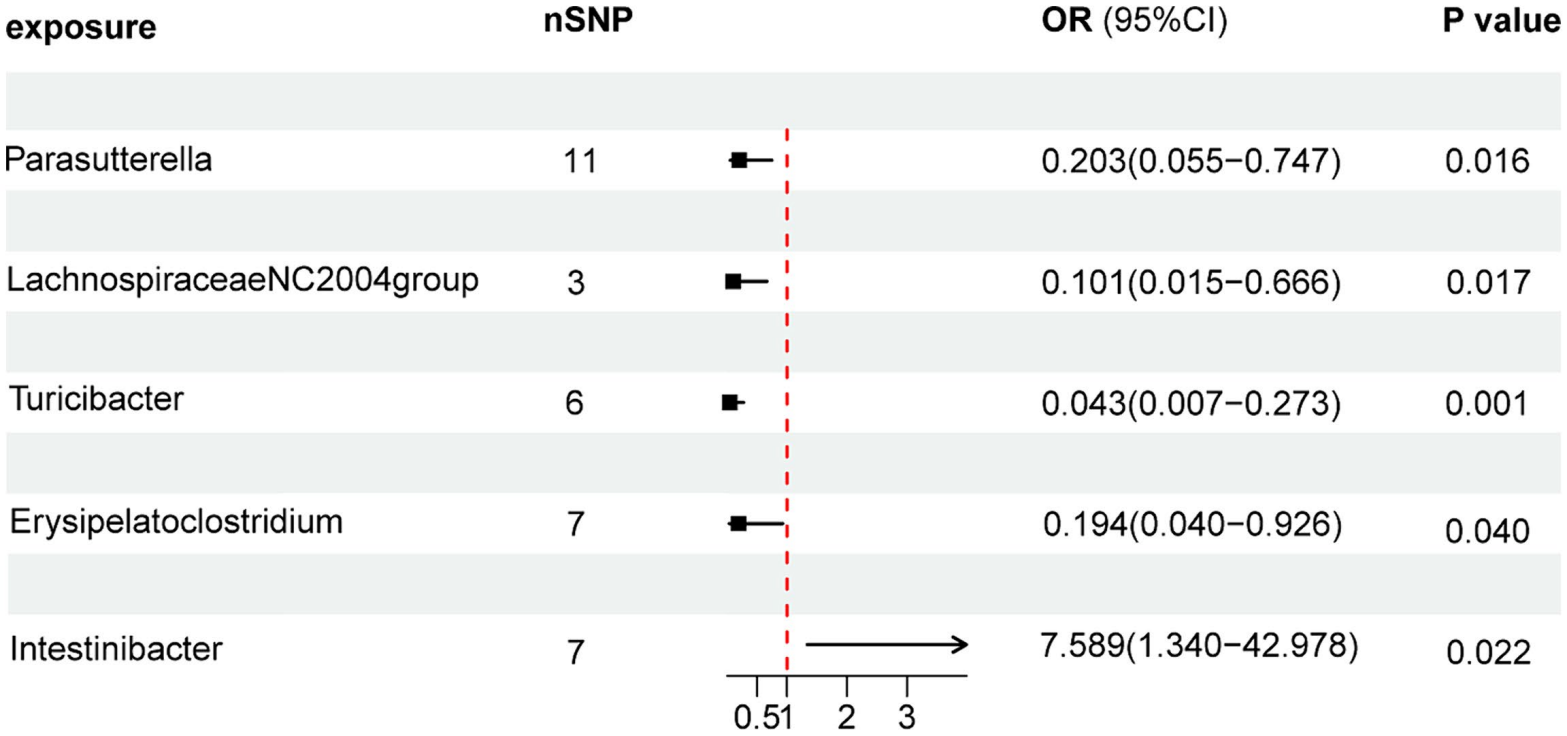
Forest plots of MR results for gut microbiota on the risk of Behçet’s disease through IVW method. MR, Mendelian Randomization; nSNP, Number of single nucleotide polymorphisms; IVW, Inverse-variance weighted; and OR, Odds ratio.

**Figure 3:**
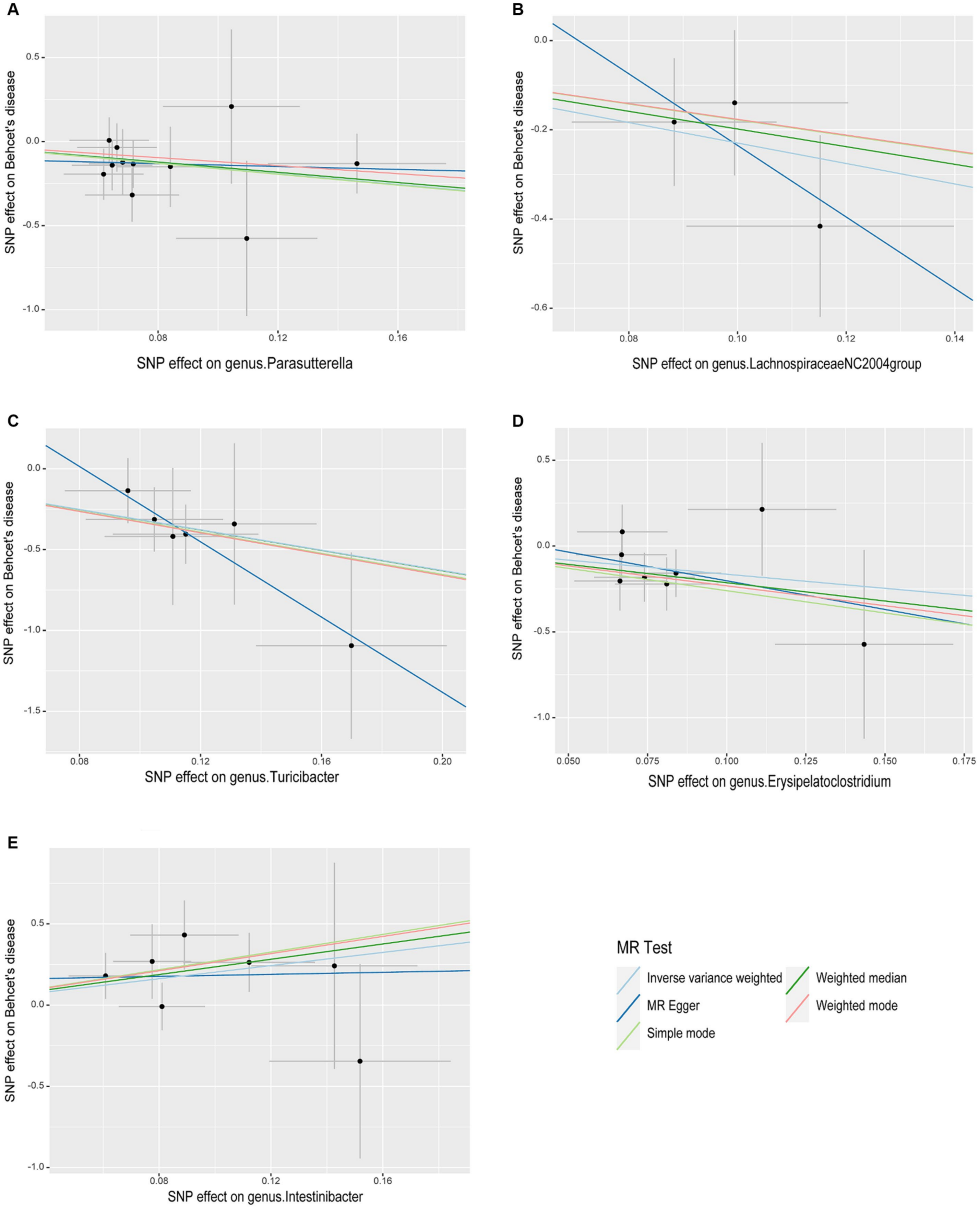
Scatter plots of causal associations of five gut microbiome taxa on Behçet’s disease. **(A)** genus. Parasutterella (id. 2892); **(B)** genus. Lachnospiraceae NC2004group (id. 11316); **(C)** genus. Turicibacter (id. 2162); **(D)** genus. Erysipelatoclostridium (id. 11381); **(E)** genus. Intestinibacter (id. 11345); MR, Mendelian randomization; SNP, Single nucleotide polymorphism.

As illustrated in Figure 2, the MR analysis results revealed that five gut microbiota had a significant association on BD. IVW results showed that the genus Parasutterella (OR = 0.203, 95%CI 0.055–0.747, *p* = 0.016), genus Lachnospiraceae NC2004 group (OR = 0.101, 95%CI 0.015–0.666, *p* = 0.017), genus Turicibacter(OR = 0.043, 95%CI 0.007–0.273, *p* = 0.001), and genus Erysipelatoclostridium (OR = 0.194, 95%CI 0.040–0.926, *p* = 0.040) were protective factors against BD, while genus Intestinibacter (OR = 7.589, 95%CI 1.340–42.978, *p* = 0.022) might be a risk factor for BD, further detailed information is available in Table 1. Figure 3 presents the scatter plot of MR analyses for five gut microbiome taxa on BD.

**Table 1:** MR analysis and sensitivity analysis of the causal effect of gut microbes on the risk of Behçet’s disease.

Exposure	Method	OR (95%CI)	*p* value	Heterogeneity test		Horizontal pleiotropy test	
				*Q*	*p*	Egger intercept	*p*
Parasutterella (nSNP = 11)	MR Egger	0.648(0.008–53.979)	0.852		0.907	−0.096	0.603
	Weighted median	0.218(0.037–1.301)	0.095				
	Inverse variance weighted	0.203(0.055–0.747)	0.016	4.754			
	Simple mode	0.198(0.016–2.390)	0.232				
	Weighted mode	0.304 (0.042–2.187)	0.264				
Lachnospiraceae NC2004group (nSNP = 3)	MR Egger	0.000 (0.000–22993.069)	0.544	0.873	0.646	0.568	0.644
	Weighted median	0.138 (0.012–1.599)	0.113				
	Inverse variance weighted	0.101 (0.015–0.666)	0.017				
	Simple mode	0.169 (0.011–2.540)	0.327				
	Weighted mode	0.171 (0.009–3.145)	0.357				
Turicibacter (nSNP = 6)	MR Egger	0.000 (0.000–13.803)	0.185		0.885	0.803	0.304
	Weighted median	0.043 (0.004–0.473)	0.010				
	Inverse variance weighted	0.043 (0.007–0.273)	0.001	1.735			
	Simple mode	0.038 (0.002–0.893)	0.098				
	Weighted mode	0.037 (0.003–0.502)	0.056				
Erysipelatoc-lostridium (nSNP = 7)	MR Egger	0.035 (0.000–858.164)	0.540			0.132	0.750
	Weighted median	0.118 (0.014–0.971)	0.047				
	Inverse variance weighted	0.194 (0.040–0.926)	0.040	3.882	0.793		
	Simple mode	0.074 (0.004–1.362)	0.123				
	Weighted mode	0.098 (0.006–1.542)	0.143				
Intestinibacter (nSNP = 7)	MR Egger	1.371 (0.001–1857.415)	0.935			0.151	0.652
	Weighted median	10.493 (1.019–108.039)	0.048				
	Inverse variance weighted	7.589 (1.340–42.978)	0.022	4.422	0.620		
	Simple mode	15.165 (0.393–585.489)	0.195				
	Weighted mode	14.033 (0.691–284.814)	0.136				

SNP, Single nucleotide polymorphism; OR, Odds ratio; CI, Confidence interval.

**Figure 4:**
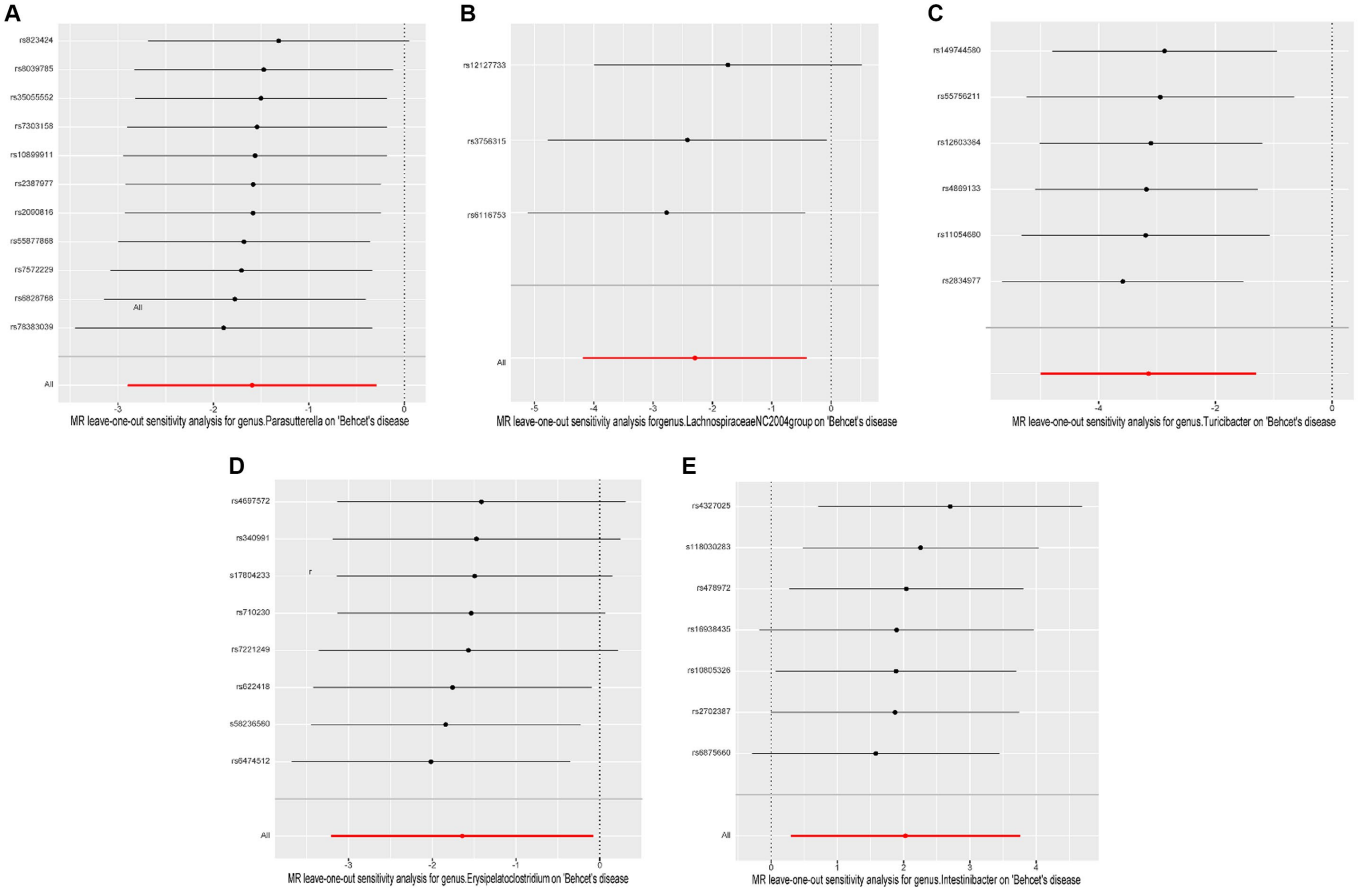
Leave-one-out plots of causal associations of five gut microbiome taxa on Behçet’s disease. **(A)** genus. Parasutterella (id. 2892); **(B)** genus. Lachnospiraceae NC2004group (id. 11316); **(C)** genus. Turicibacter (id. 2162); **(D)** genus. Erysipelatoclostridium (id. 11381); **(E)** genus. Intestinibacter (id. 11345); MR, Mendelian randomization.

Cochrane’s *Q* test of the IVW methods revealed all *p* values to be >0.05, suggesting the absence of heterogeneity. Additionally, the MR-Egger intercept test indicated no evidence of potential horizontal pleiotropy (*p* > 0.05). The detailed results are summarized in Table 1. The leave-one-out analysis results for the association between gut microbiota and BD are presented in Figure 4. Excluding SNPs one by one, all instrumental variables were on the side of 0, indicating the robustness of the MR analysis results.

### 3.2 Causal effects of BD on gut microbiota

Finally, we conducted a reverse MR analysis to assess the potential reverse causal relationships between the genera Parasutterella, Lachnospiraceae NC2004 group, Turicibacter, Erysipelatoclostridium, and Intestinibacter with BD. The IVW results revealed that there was no significant association between BD and any of the five genera: Parasutterella (OR = 0.990, 95%CI 0.973–1.007, *p* = 0.256), Lachnospiraceae NC2004 group (OR = 1.007, 95%CI 0.976–1.039, *p* = 0.665), Turicibacter (OR = 1.009, 95%CI 0.989–1.028, *p* = 0.383), Erysipelatoclostridium (OR = 0.983, 95%CI 0.964–1.002, *p* = 0.082), and Intestinibacter (OR = 0.993, 95%CI 0.977–1.009, *p* = 0.374). The reverse MR analysis exhibited no heterogeneity (*p* > 0.05) or pleiotropy (p > 0.05), indicating robust results, as detailed in Table 2.

**Table 2:** MR analysis and sensitivity analysis of the causal effect for Behçet’s disease on gut microbes.

Outcome	Method	OR (95%CI)	*p* value	Heterogeneity test		Horizontal pleiotropy test	
				*Q*	*p*	Egger intercept	*p*
Parasutterella (nSNP = 3)	MR Egger	1.018 (0.956–1.085)	0.672		0.641	−0.030	0.530
	Weighted median	0.988 (0.968–1.009)	0.260				
	Inverse variance weighted	0.990 (0.973–1.007)	0.256	0.888			
	Simple mode	0.983 (0.957–1.009)	0.327				
	Weighted mode	0.983 (0.956–1.010)	0.345				
Lachnospiraceae NC2004group (nSNP = 3)	MR Egger	0.934 (0.857–1.019)	0.367	3.512	0.173	0.078	0.329
	Weighted median	1.015 (0.984–1.047)	0.339				
	Inverse variance weighted	1.007 (0.976–1.039)	0.665				
	Simple mode	1.027 (0.983–1.072)	0.357				
	Weighted mode	1.026 (0.977–1.078)	0.407				
Turicibacter (nSNP = 3)	MR Egger	1.026 (0.955–1.101)	0.612		0.854	−0.018	0.716
	Weighted median	1.011 (0.988–1.035)	0.340				
	Inverse variance weighted	1.009 (0.989–1.028)	0.383	0.316			
	Simple mode	1.012 (0.983–1.041)	0.506				
	Weighted mode	1.012 (0.986–1.038)	0.468				
Erysipelatoc-lostridium (nSNP = 3)	MR Egger	1.032 (0.964–1.104)	0.531			−0.051	0.383
	Weighted median	0.982 (0.960–1.004)	0.114				
	Inverse variance weighted	0.983 (0.964–1.002)	0.082	2.1632	0.339		
	Simple mode	0.971 (0.940–1.004)	0.225				
	Weighted mode	0.972 (0.941–1.004)	0.231				
Intestinibacter (nSNP = 4)	MR Egger	1.037 (0.980–1.098)	0.334			−0.047	0.255
	Weighted median	0.999 (0.978–1.020)	0.901				
	Inverse variance weighted	0.993 (0.977–1.009)	0.374	2.683	0.443		
	Simple mode	1.001 (0.972–1.031)	0.954				
	Weighted mode	1.002 (0.979–1.026)	0.861				

SNP, Single nucleotide polymorphism; OR, Odds ratio; CI, Confidence interval.

## 4 Discussion

This study utilized publicly available GWAS summary statistics and employed the two-sample MR method to investigate the causal relationship between 211 gut microbiota and BD. The findings suggest that five bacterial taxa may have a causal relationship with BD risk. Specifically, an increase in the abundance of the genus Parasutterella, Lachnospiraceae NC2004 group, Turicibacter, and Erysipelatoclostridium was associated with a decreased risk of BD, while an increase in the abundance of the genus Intestinibacter was linked to an elevated risk of BD.

Behçet’s disease is a rare immune-mediated neutrophilic vasculitis characterized by multi-organ involvement, with autoimmune dysregulation as a potential mechanism of pathogenesis (Sakane et al., 1999). Abnormal activities of T helper cell 1 (Th1), Th17, and regulatory T cells (Treg) has been observed in BD patients, with the Th17/Tregs ratio being significantly higher in BD patients compared to healthy controls (Wilson et al., 2007; Takeuchi et al., 2015; Shimizu et al., 2016b; Ahmadi et al., 2019). Th17 cells are considered to be the highly pathogenic T cells that mediate inflammatory responses and autoimmune diseases, including BD (Hamzaoui et al., 2011; Na et al., 2013). Therefore, the pathogenesis of BD may result from an immune tolerance defect due to a decrease in Tregs, while the increase in Th17 cells triggers inflammation (Ma et al., 2021). Recent research has confirmed that changes in gut microbiota are involved in the occurrence of BD by regulating Th1, Th17, and Treg cells.

In many autoimmune diseases, changes in the composition of the gut microbiota have been observed, including rheumatoid arthritis (RA) (Brandl et al., 2021), systemic lupus erythematosus (SLE) (He et al., 2016), inflammatory bowel disease (IBD) (Paramsothy et al., 2019), and Type 1 Diabetes (T1DM) (Del Chierico et al., 2022). Similarly, traditional observational studies have reported an association between dysbiosis of gut microbiota and BD. Consolandi and associates first reported the gut microbiota characteristics in BD. Compared to healthy controls, Behçet’s patients showed significant depletion in the genera Roseburia and Subdoligranulum, and significantly reduced levels of butyrate production (*p* = 0.0033) (Consolandi et al., 2015). Shimizu et al. (2016a) reported a significant increase in the relative abundance of Bifidobacterium and Eggerthella, and decrease in the relative abundance of Megamonas and Prevotella in BD patients, in a gut microbiome study of 12 Japanese patients with BD and 12 healthy controls. These previous cross-sectional studies have found changes in the abundance of gut microbiota in patients with BD and have reached a consistent conclusion: the gut microbiota of BD patients is rich in lactic acid-producing bacteria, sulfate-reducing bacteria, and some opportunistic pathogens, but lacks butyric acid-producing bacteria and methanogenic bacteria (Sun et al., 2022).

The alterations in microbial-derived metabolites represent an important mechanism through which changes in gut microbiota can impact host health. For instance, short-chain fatty acids (SCFAs), as a key group of metabolites produced by gut microbiota, play a crucial role in connecting diet, gut microbiota, and host immune responses. The primary SCFAs produced by gut bacteria are acetate, propionate, and butyrate, with the highest levels found in butyrate (Kim et al., 2014; Golpour et al., 2023). Many bacteria that produce SCFAs are reduced in systemic autoimmune diseases. However, the causal relationship between changes in gut microbiota and BD remains incompletely understood. In order to determine the precise role of the gut microbiota in the development of BD, researchers conducted fecal transplantation in mice with autoimmune uveitis. They found that mice colonized with the whole gut microbiome from BD patients often exhibited an exacerbation of disease activity and excessive production of pro-inflammatory cytokines (Ye et al., 2018). In addition, a study on the role of gut microbiota in Behcet’s uveitis and Vogt-Koyanagi-Harada syndrome revealed the disease-specific composition of gut microbiota in Behçet’s uveitis and demonstrated that the gut microbiota may promote the onset of the disease through the cumulative effects of microbial antigens and their metabolites (Wang et al., 2023).

The Lachnospiraceae NC2004 group is a member of the Lachnospiraceae family, belonging to the Firmicutes phylum. The Lachnospiraceae family is composed of strict anaerobic bacteria, forming the core of the gut microbiota and being one of the most abundant bacteria in the human intestinal tract (Vacca et al., 2020). The reduction of Lachnospiraceae in the gut microbiota has been associated with a range of diseases. In a cross-sectional study, Oezguen et al. (2019) discovered that Unclassified Lachnospiraceae was decreased in BD patients compared to healthy individuals. This study supports the notion. We found that Lachnospiraceae NC2004 group is a protective factor for BD. A plausible hypothesis is that the Lachnospiraceae family regulates BD symptoms by producing butyrate. It is well known that butyrate promotes the differentiation and function of Tregs and limits inflammatory Th17 cells to improve the inflammatory response (Furusawa et al., 2013; Koh et al., 2016), deficiencies in butyrate production may lead to reduced Treg responses and activation of immunopathological T effector responses. Additionally, our research also found that increased abundance of the genus Erysipelatoclostridium, Parasutterella, and Turicibacter has a protective effect against BD. Erysipelatoclostridium and Turicibacter were also classified as beneficial bacteria that promote the production of SCFAs (Beisner et al., 2020; Liu et al., 2022) and Parasutterella is capable of produce succinate to support the cross-feeding within gut microbiota (Fischbach and Sonnenburg, 2011; Ju et al., 2019).

Intestinibacter bartlettii, previously known as *Clostridium bartlettii*, has been reclassified. Observational studies have suggested a significant increase in the abundance of Intestinibacter in patient with Moyamoya disease (MMD) and neurodevelopmental disorders (NDD) (Bojović et al., 2020; Mineharu et al., 2022). Additionally, metformin can alter the composition of the gut microbiota by reducing the levels of Intestinibacter (Rodriguez et al., 2018). Hu et al. found that patients with IgA vasculitis had higher levels of the genus Intestinibacter compared to healthy controls (Hu et al., 2022). Interestingly, this study, using MR methods for the first time, suggests that an increased abundance of Intestinibacter may elevate the risk of BD, but the underlying mechanism remains to be fully elucidated.

The gut microbiota can participate in immune and inflammatory responses through various ways, such as the production of metabolites and microbial components like lipopolysaccharides, direct generation of immune mediators, and influence on intestinal barrier function (Chen and Tang, 2023). Changes in the gut microbiota of patients with BD and the subsequent impairment of SCFAs production, decreased expression of tight junction proteins, reduced production of mucins (MUCs), and antimicrobial peptides, can lead to the breakdown of the intestinal barrier. This allows effector molecules or pathogen-associated molecular patterns (PAMPs) to migrate into the blood and adjacent organs and tissues, triggering vasculitis and tissue damage, and promoting systemic inflammation (Cheng et al., 2023). The chronic inflammatory state of BD may lead to alterations in the local microbial composition, inducing disruption of mucosal barrier function and resulting in various local adverse reactions such as oral ulcers, genital ulcers, skin and ocular manifestations (Ogunkolade et al., 2023). However, this hypothesis requires further in-depth research for validation in the future.

The human gut microbiota is composed of approximately 100 trillion resident microorganisms, with bacteria being the most abundant. As a result, attention is primarily focused on bacteria. However, viruses and fungi also play an indispensable role in maintaining the balance of the intestinal environment and host health (Joubert et al., 2023). The etiological hypothesis of BD was first proposed by Dr. Hulusi Behcet in 1937. Many viral infections may be associated with BD, such as herpes simplex virus (HSV), hepatitis C viruses (HCV), hepatitis B viruses (HBV), Parvovirus B19, and human immunodeficiency virus (HIV), but conclusive evidence is lacking (Sciascia et al., 2023). The main component of the gut virome is bacteriophage. Bacteriophages can infect and replicate inside host bacteria, not only controlling the abundance of gut bacteria through lytic infection but also influencing immune system regulation by directly interacting with immune cells (Zuppi et al., 2021). It has been shown that the composition of gut virome is significantly different in patients with IBD compared to healthy controls (Norman et al., 2015). In addition, Tomofuji et al. (2022) discovered a significant reduction of crAss-like bacteriophages in the gut of certain autoimmune disease patients, particularly those with RA and SLE, through gut virome analysis, further elucidating the connection between bacteriophages and autoimmune diseases. Fungal infections can trigger various systemic diseases, and gut fungi represent a small yet crucial component of the human gut microbiome. Research has revealed significant changes in the gut fungal composition of patients with certain autoimmune diseases compared to healthy controls, for instance, the abundance of *Candida albicans* significantly increases in patients with asthma, IBD, and multiple sclerosis (Yadav et al., 2022; Zhang et al., 2022). The potential mechanism is that the colonization of *Candida albicans* may drive Th17-mediated inflammatory responses or indirectly impact the host’s immune homeostasis through interactions with bacteria, exacerbating the onset of disease (Zhang et al., 2022). Therefore, we have reason to speculate that the composition of the gut virome and fungi may play vital roles in promoting or alleviating inflammatory BD.

Our study has clinical implications providing significant insights into the causal relationship between the human gut microbiome and the development of BD. It also offers important scientific evidence for the potential use of the gut microbiome as a tool for the prevention, diagnosis, and treatment of BD. The high abundance of the genera Parasutterella, Lachnospiraceae NC2004 group, Turicibacter, Erysipelatoclostridium may reduce the risk of BD, and this finding could be applied to the research and development of novel probiotic/prebiotic supplements aimed at preventing the onset of diseases. Additionally, it suggests that by intervening in the diets and lifestyles of BD patients, we may assist in restoring the equilibrium of immune system (Pagliai et al., 2020). Conversely, the high abundance of the genus Intestinibacter may increase the risk of BD, indicating that the elevation in the concentration of this bacterial group could serve as a potential indicator for evaluating the risk of BD, thus providing valuable guidance for clinical applications. In the future, manipulating the gut microbiota may serve as an intervention that can not only help improve clinical symptoms for patients with active BD but also aid in preventing disease flares in those in remission. Furthermore, it may prevent disease onset in patients with a familiar/genetic predisposition to BD susceptibility (Bettiol et al., 2023).

Our study represents the first large-scale MR analysis aimed at assessing the causal relationship between the gut microbiome and BD at the genetic level. However, our study has some limitations. Firstly, the majority of GWAS participants were of European descent, which may restrict the generalization of our findings to other ethnic groups. Secondly, although Europeans comprise the majority (nearly 80%), the GWAS data on gut microbiome are predominantly from multiple populations, which may introduce some confounding factors; nevertheless, it has been used widely in convincing studies for MR analysis. Further research on data from the same population may be needed in the future. Thirdly, the original gut microbiome studies lack species-level GWAS summary statistics. Hence, we were unable to identify taxonomic groups at the species level that are causally related to BD. Finally, in our MR analysis, there were cases of inflated OR values in some MR-Egger results, potentially due to small sample sizes or the influence of confounding factors. Though this does not fundamentally affect the study’s conclusions, as we primarily focus on the IVW results, we still hope for more in-depth research using additional GWAS data in the future.

## 5 Conclusion

We investigated the causal relationship between the gut microbiome and BD through a two-sample MR method, using publicly available GWAS summary data. We identified one potential pathogenic bacterium and four beneficial probiotic taxonomic groups that influence the onset of BD. This provides valuable biomarkers for the prevention, early diagnosis, and potential therapeutic targets for BD.

## Data availability statement

Publicly available datasets were analyzed in this study. The names of the repository/repositories and accession number(s) can be found below: Behcet’s disease: ieu open GWAS project (ebi-a-GCST90018798; https://gwas.mrcieu.ac.uk/); Gut microbiota: MiBioGen consortium (https://mibiogen.gcc.rug.nl/).

## Ethics statement

Ethical approval was not required for the studies involving humans because this study is based on large-scale GWAS datasets, and not individual-level data. Ethical approval is not applicable. The studies were conducted in accordance with the local legislation and institutional requirements. Written informed consent for participation was not required from the participants or the participants’ legal guardians/next of kin in accordance with the national legislation and institutional requirements.

## Author contributions

Y-NL: Methodology, Writing – original draft, Writing – review & editing. TC: Writing – original draft. YX: Writing – original draft. J-YJ: Writing – original draft. T-KY: Writing – original draft. P-CX: Funding acquisition, Writing – original draft, Writing – review & editing, Conceptualization, Validation.
